# Functional Characterization of CLPTM1L as a Lung Cancer Risk Candidate Gene in the 5p15.33 Locus

**DOI:** 10.1371/journal.pone.0036116

**Published:** 2012-06-04

**Authors:** Michael A. James, Weidong Wen, Yian Wang, Lauren A. Byers, John V. Heymach, Kevin R. Coombes, Luc Girard, John Minna, Ming You

**Affiliations:** 1 MCW Cancer Center, Department of Pharmacology and Toxicology, Medical College of Wisconsin, Milwaukee, Wisconsin, United States of America; 2 Department of Surgery, Washington University School of Medicine, St. Louis, Missouri, United States of America; 3 The University of Texas MD Anderson Cancer Center, Houston, Texas, United States of America; 4 Hamon Center for Therapeutic Oncology Research, Simmons Cancer Center, Department of Pharmacology, University of Texas Southwestern Medical Center at Dallas, Dallas, Texas, United States of America; Virginia Commonwealth University, United States of America

## Abstract

Cleft Lip and Palate Transmembrane Protein 1-Like (CLPTM1L), resides in a region of chromosome 5 for which copy number gain has been found to be the most frequent genetic event in the early stages of non-small cell lung cancer (NSCLC). This locus has been found by multiple genome wide association studies to be associated with lung cancer in both smokers and non-smokers. CLPTM1L has been identified as an overexpressed protein in human ovarian tumor cell lines that are resistant to cisplatin, which is the only insight thus far into the function of CLPTM1L. Here we find CLPTM1L expression to be increased in lung adenocarcinomas compared to matched normal lung tissues and in lung tumor cell lines by mechanisms not exclusive to copy number gain. Upon loss of CLPTM1L accumulation in lung tumor cells, cisplatin and camptothecin induced apoptosis were increased in direct proportion to the level of CLPTM1L knockdown. Bcl-xL accumulation was significantly decreased upon loss of CLPTM1L. Expression of exogenous Bcl-xL abolished sensitization to apoptotic killing with CLPTM1L knockdown. These results demonstrate that CLPTM1L, an overexpressed protein in lung tumor cells, protects from genotoxic stress induced apoptosis through regulation of Bcl-xL. Thus, this study implicates anti-apoptotic CLPTM1L function as a potential mechanism of susceptibility to lung tumorigenesis and resistance to chemotherapy.

## Introduction

CLPTM1L is so named based on its homology with Cleft Lip and Palate Transmembrane Protein 1, which was identified as disrupted in a family with cleft lip and palate [1]. CLPTM1L was identified as an up-regulated transcript in a cisplatin resistant ovarian tumor cell line [2]. However, interpretation of the results of this study is difficult, as there is no implication of mechanism and the effect of overexpression of CLPTM1L in cisplatin sensitivity was conflicting in different ovarian tumor cell lines, depending on their pre-existing level of resistance. Nevertheless, a role for CLPTM1L in resistance to cisplatin was suggested. Interestingly, the homologue CLPTM1 has been found to be expressed at higher levels in doxorubicin resistant breast tumors, and expression of CLPTM1 is predictive of response to doxorubicin [3]. A recent study found that a genetic variant within the CLPTM1L gene (rs402710) is associated with the accumulation of DNA adducts in tumor adjacent lung tissue [4]. This same SNP, among others in the region of the CLPTM1L and TERT genes is associated with risk of lung cancer [5], [6], [7]. In a recent study on cervical cancer integrating gene dosage and expression data, the CLPTM1L/TERT locus was found to have copy number gain in tumors and expression patterns that correlated with copy number gain [8]. Another recent study revealed that with copy number gain across 5p, CLPTM1L expression was increased approximately 5 fold in cervical cancer cell lines over normal cervical epithelial cells, while expression of the other genes at 5p15.33 was not changed [9]. These insights into the function of CLPTM1L, and the fact that copy number gain of the region of chromosome 5p containing CLPTM1L is the most frequent cytogenetic event in the early stages of non-small cell lung cancer (NSCLC) [10] are compelling justification for the study of the role of CLPTM1L in lung cancer as well as other cancer types.

DNA damage, such as that caused by genotoxic chemotherapeutic agents, induces apoptosis through double stranded break associated kinases, and subsequent transcriptional regulation of apoptotic effectors primarily through p53 [11]. Bcl-2 family members regulated by p53 including Bax are central to the activation of apoptosis by this pathway and act by permeabilizing the mitochondrial membrane [12]. Anti-apoptotic Bcl-2 family member Bcl-xL protects cancer cells from p53 induced apoptosis [13] and acts through the binding and inactivation of Bax [14] and binding of proteins that recruit Bax to the mitochondrial membrane [15]. Bcl-xL is frequently overexpressed in lung tumors, is associated with poor prognosis [16], [17] and plays an important role in resistance to genotoxic chemotherapeutic agents in lung and other cancer types [18], [19], [20], [21], [22], [23].

Although a connection of CLPTM1L to cancer is suggested by copy number gain, genome wide association and studies in ovarian tumor cell lines; the function of CLPTM1L and its role in tumorigenesis is thus far unknown. Here we report that CLPTM1L is a commonly overexpressed anti-apoptotic factor in lung tumors. Knockdown of CLPTM1L transcript in NSCLC cells results in an increase in sensitivity to genotoxic stress mediated apoptotic killing and diminishes expression of Bcl-xL in a manner dependent on the dose of CLPTM1L expression. Moreover, expression of exogenous Bcl-xL abolishes sensitization to genotoxic stress induced apoptosis by CLPTM1L knockdown. This protective effect is not exclusive to cisplatin mediated killing. Rather, CLPTM1L acts indirectly as a general inhibitor of the mitochondrial pathway of apoptosis through Bcl-xL regulation. These results demonstrate a role for CLPTM1L in chemotherapeutic resistance in lung tumor cells, and suggest a role for CLPTM1L in lung tumorigenesis via protection from apoptosis through increased accumulation of Bcl-xL.

## Results

### Expression of CLPTM1L in Lung Tumors

**Figure 1:**
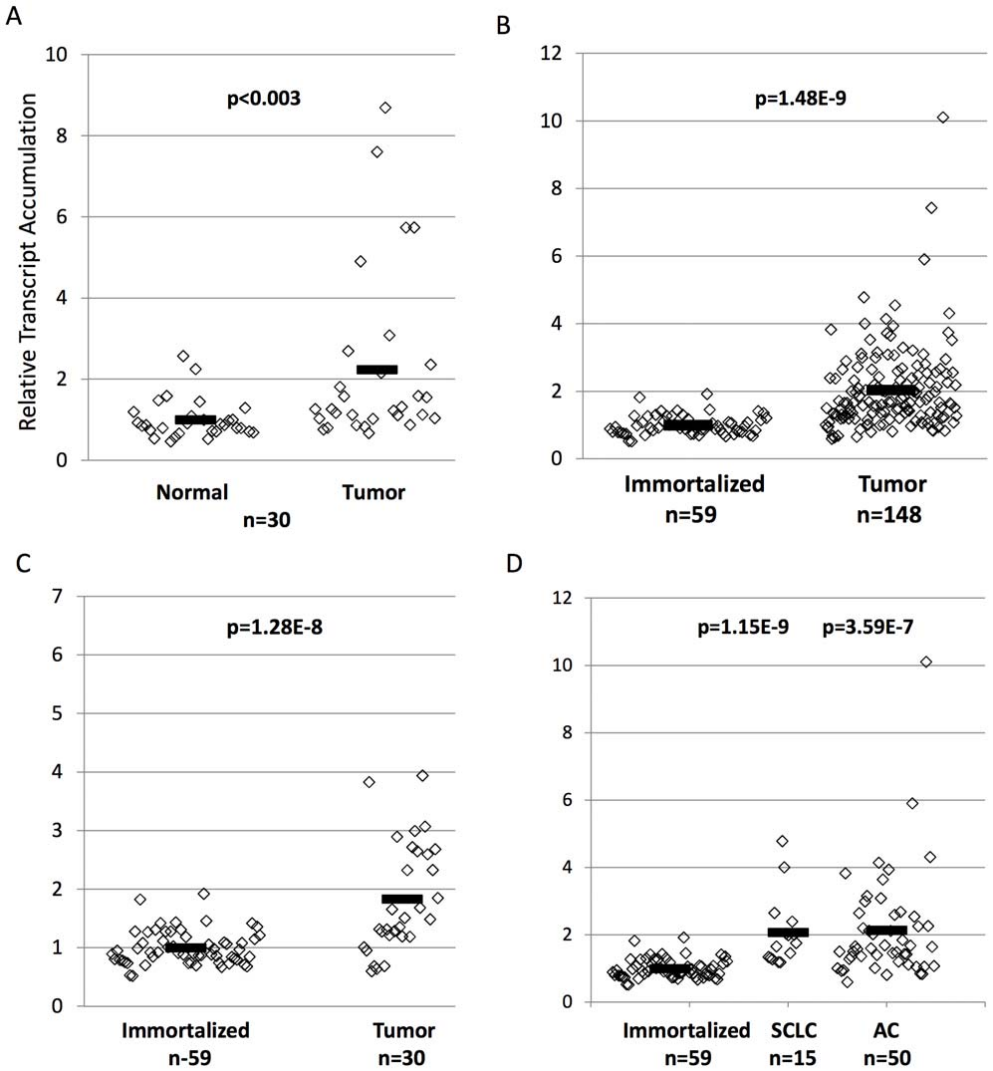
Expression of CLPTM1L is increased in lung adenocarcinomas and in lung tumor cell lines. A) CLPTM1L transcript accumulation as measured by qPCR in lung adenocarcinoma tissues relative to the mean of matched normal tumor adjacent tissue in 30 patients demonstrating a 2.23 fold average increase in expression in tumor tissues. B) CLPTM1L transcript accumulation as measured by microarray in lung tumor cell lines relative to the mean of non-transformed immortalized cell lines demonstrating a 2.02 fold average increase in expression in tumor cell lines. C) Cell line expression data excluding those tumor cell lines with copy number variation demonstrating a 1.83 fold increase in tumor cell lines. D) Cell line divided into adenocarcinoma cell lines and small cell lung cancer cell lines. Black bars represent average values. p-values were obtained using a two-tailed Student’s T-Test.

Given reports of copy number gain in lung cancer, GWAS evidence, and increased expression of CLPTM1L in cisplatin resistant ovarian tumor cells, we sought to determine if CLPTM1L was overexpressed in lung tumors. Expression of CLPTM1L mRNA in NSCLC patient tumors was compared to that of matched tumor-adjacent tissues by qPCR. CLPTM1L was increased by an average of 2.24 fold reaching an overall significance for differential expression in 30 Stage I NSCLC patients (p  = .0028, Two-tailed Student’s T-Test) (Figure 1A). Although TERT expression was an average of 1.76 fold higher in tumor tissues, overall differences in TERT expression did not reach significance and were not significantly correlated with CLPTM1L expression (r^2^ = 0.0018, p = 0.994) (Figure S1A). The sample consisted of 22 adenocarcinoma and 8 squamous cell carcinoma patients Table S1 describes the known characteristics of the study population. There was no difference in tumor over-expression between squamous cell carcinomas and adenocarcinomas (data not shown). Analysis of expression microarray data from 148 lung tumor cell lines and 59 “normal” cell lines immortalized with TERT and Cdk4 confirmed these results, revealing a 2.02 fold average difference in CLPTM1L expression compared to immortalized cell lines (p  = 1.48E-9, Two-tailed Student’s T-Test) (Figure 1B). This data also demonstrates that expression of CLPTM1L is increased in tumor cells by mechanisms other than copy number variation, as analysis excluding those tumor cell lines with copy number variation remained significant (p  = 1.28E-8, Two-tailed Student’s T-Test) and similar in magnitude (1.83 fold) (Figure 1C). Copy number change was identical between CLPTM1L and TERT genes. However, no correlation between CLPTM1L expression and TERT expression was observed within tumor cell lines (r^2^ = 0.0126, p = 0.175) (Figure S1B). Analysis of expression was also conducted for different lung tumor subtypes. Adenocarcinoma cell lines showed an average 2.15 fold greater CLPTM1L expression over normal immortalized cell lines (p = 3.59E-7, Two-tailed Student’s T-Test) and Small Cell Lung Cancer cell lines showed an average 2.07 fold greater expression (p = 1.15E-9, Two-tailed Student’s T-Test) (Figure 1D). Therefore, increased CLPTM1L expression appears to be a feature of lung tumors regardless of subtype.

### CLPTM1L Confers Resistance to Genotoxic Stress Induced Apoptosis in Lung Tumor Cells

**Figure 2:**
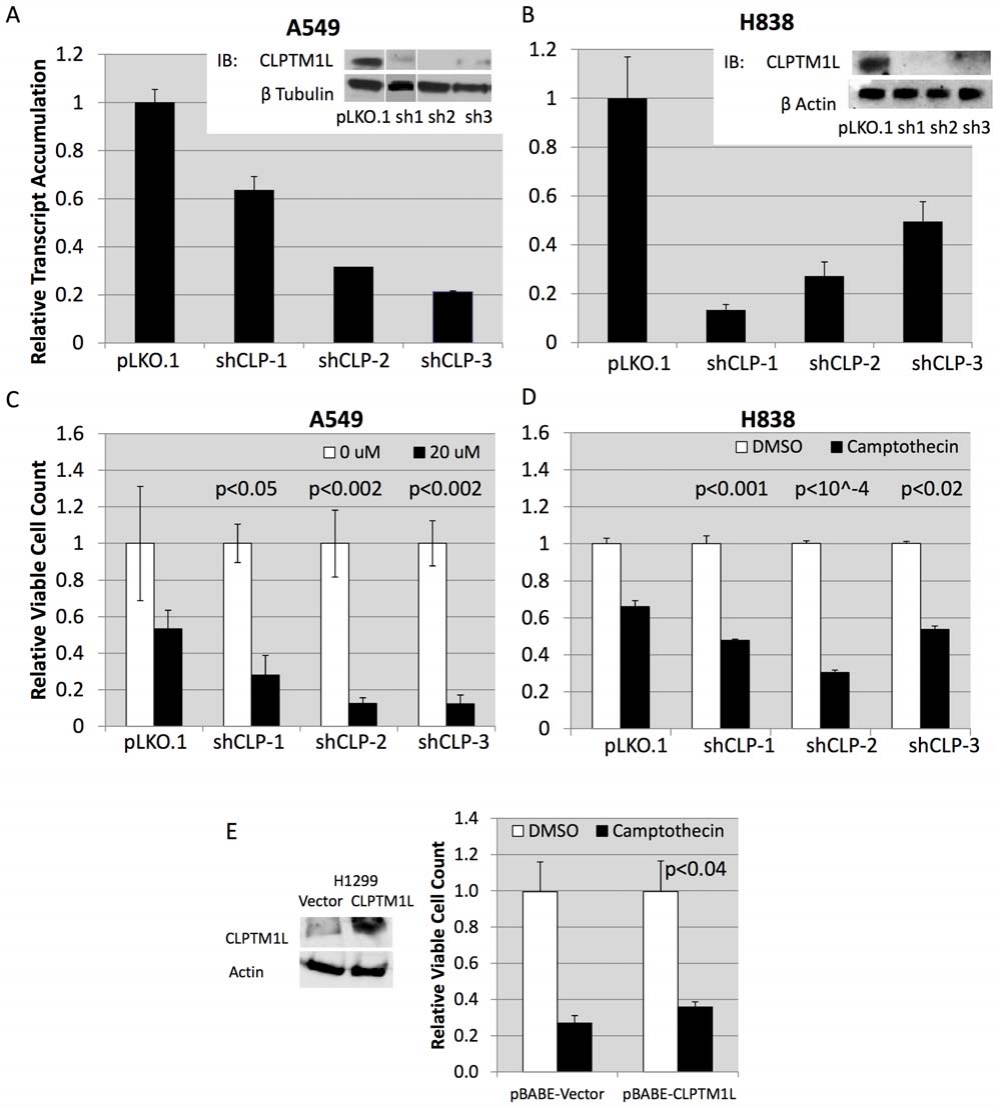
Loss of CLPTM1L sensitizes lung tumor cells to genotoxic agents. A) Knockdown of CLPTM1L expression in A549 cells via stable shRNA. Confirmation of knockdown by western blot (inset). B) Knockdown of CLPTM1L expression in H838 cells via stable shRNA. Confirmation of knockdown by western blot (inset). C) Relative cell viability of A549 cells with CLPTM1L knockdown after 48 hours treatment with media or cisplatin. D) Relative cell viability of H838 cells with CLPTM1L knockdown after 48 hours treatment with DMSO vehicle or 1****µM camptothecin. E) Relative cell viability of H1299 cells with CLPTM1L over-expression after 48 hours treatment with DMSO vehicle or 10****µM camptothecin. Error bars represent one standard deviation from the mean of biological triplicates.

Since increased expression of CLPTM1L is associated with lung tumors, we aimed to modulate CLPTM1L levels in the lung adenocarcinoma cell lines and determine the response to genotoxic agents. Knockdown of CLPTM1L in A549 and H838 lung adenocarcinoma cell lines was performed using three independent viral shRNA vectors and resulted in a range of efficiencies up to 90% as measured by quantitative real-time PCR and by immunoblot (Figure 2A and B). The effect of CLPTM1L knockdown on killing by cisplatin was determined in these cell lines. Cells were counted, plated in equal numbers at approximately 50% confluence, and assayed for viability by cell counting after 48 hours of cisplatin treatment. Killing of lung tumor cells by cisplatin was increased upon loss of CLPTM1L in a dose dependent manner, ranging from 53% viability in A549 cells with vector alone to 12% viability in cells with shCLPTM1L-3 (Figure 2C). Similarly, we induced apoptosis in A549 cells with stable knockdown of CLPTM1L using camptothecin, a topoisomerase I inhibitor A549 cells were plated in equal numbers and assayed for viability by MTS assay after 48 hours of treatment with camptothecin. Again, loss of CLPTM1L through shRNA knockdown sensitized lung tumor cells to dose dependent apoptotic killing in a manner consistent with the level of CLPTM1L expression (Figure S2), demonstrating that the anti-apoptotic effects of CLPTM1L is not exclusive to cisplatin. Similar results were observed in H838 cells, which demonstrated increased sensitivity to camptothecin induced killing upon knockdown of CLPTM1L expression (Figure 2D). Consistent with these findings, exogenous over-expression of CLPTM1L in H1299 cells, which were determined to express relatively low levels of endogenous CLPTM1L transcript compared to A549 cells by microarray analysis (data not shown), resulted an increase in cell viability from 27% with vector alone to 36% with CLPTM1L over-expression (p>0.04) after treatment with camptothecin relative to DSMO solvent controls (Figure 2E).

**Figure 3:**
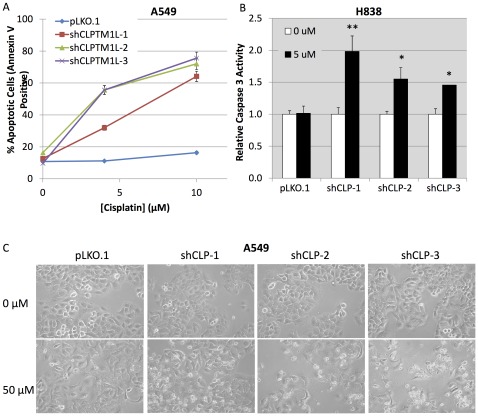
DNA damage induced apoptosis is regulated by CLPTM1L. A) Annexin V binding by flow cytometry of A549 cells with CLPTM1L knockdown after 48 hours treatment with cisplatin. B) Relative caspase 3/7 activity in H838 cells with CLPTM1L knockdown after 48 hours treatment with cisplatin. Error bars represent one standard deviation from the mean. **- p<0.01 * - p<0.02 by two-tailed Student’s T-Test. C) Micrographs of A549 cells with CLPTM1L knockdown after 24 hours treatment with 50****µM cisplatin showing increased genotoxic cell death upon loss of CLPTM1L.

Annexin V immunofluorescence detected by flow cytometry was used as a marker of early apoptosis in A549 cells with or without CLPTM1L knockdown and cisplatin treatment. We observed a dose dependent increase in apoptosis with loss of CLPTM1L (Figure 3A). While only 16% of cells with vector alone were apoptotic after 10µM cisplatin treatment for 24 hours, 76% of cells with shCLPTM1L-3 were apoptotic. Resistance to cisplatin induced apoptosis was found to be proportional to the amount of CLPTM1L transcript accumulation in these cells, with an r^2^ of 0.9808 (p = 2×10^−8^) between percent knockdown and percent apoptotic cells (Figure S3A). Cisplatin concentrations at the lower limits of sensitivity were used to allow resolution of sensitivities conferred by different levels of CLPTM1L knockdown. The DNA damaging agents cisplatin and nitrosamine 4-(methyl-nitrosamino)-1-(3-pyridyl)-1-butanone (NNK) both caused accumulation of DNA strand breaks independently of CLPTM1L expression as detected by the COMET method (Figure S4). Therefore, the observed increase in sensitivity tositivity to cisplatin upon loss of CLPTM1L is due to an effect on apoptosis or apoptotic signaling rather than an effect on levels of acute DNA damage. Sensitivity to cisplatin induced apoptosis was also increased with loss of CLPTM1L expression by shRNA in H838 tumor cells (Figure 3B), measured by colorimetric Caspase 3/7 activity assay. Again resistance to cisplatin induced apoptosis was proportional to the amount of CLPTM1L transcript accumulation in the cells, with an r^2^ of 0.8847 (p = 1.3×10^−4^) between percent knockdown and relative caspase 3 activity (Figure S3B). The appearance of cells in culture is consistent with increased sensitivity to cisplatin induced apoptosis upon loss of CLPTM1L (Figure 3C).

### Regulation of Bcl-xL Expression is Essential for CLPTM1L Effects on Apoptosis

**Figure 4:**
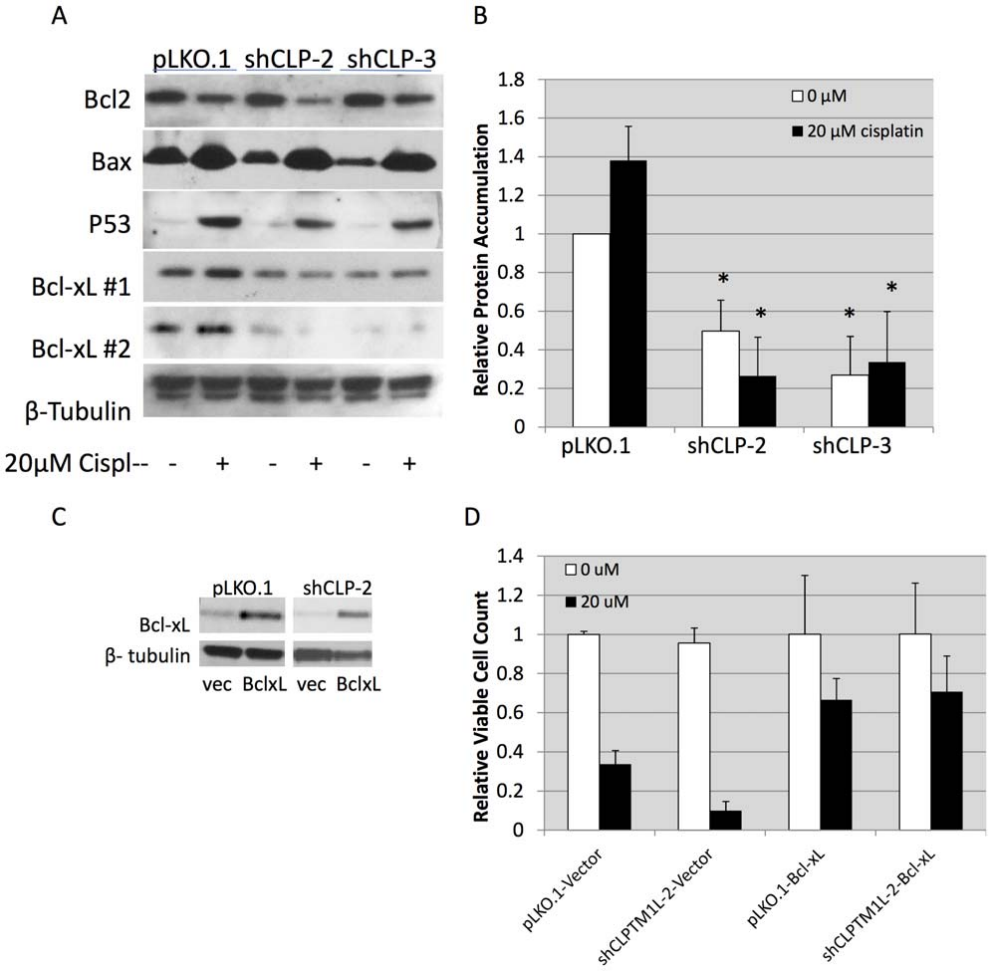
Modulation of Bcl-xL expression is required for apoptotic effects of CLPTM1L. A) Western blotting for apoptotic regulators in A549 cells with CLPTM1L knockdown after treatment with cisplatin showing decreased Bcl-xL expression upon loss of CLPTM1L. Bcl-xL blots for two separate representative clonal populations of A549 cells with CLPTM1L knockdown (#1 and #2) are shown. B) Graphic representation of Bcl-xL expression in cells with CLPTM1L knockdown from three separate clonal populationsA549. Error bars represent one standard deviation from the mean. * - p<0.003 by Student’s T-Test C) Western blots confirming expression of exogenous Bcl-xL in A549 cells with CLPTM1L knockdown. D) Relative viable cell counts in A549 cells with CLPTM1L knockdown and ectopic Bcl-xL expression after treatment with cisplatin demonstrating the abolition of apoptotic effect of CLPTM1L loss upon ectopic Bcl-xL expression.

To investigate the mechanism of CLPTM1L protection from apoptosis, we measured protein levels of apoptotic regulators in untreated A549 cells compared to those treated with cisplatin. For evaluation of the requirement of CLPTM1L for regulation of apoptosis, the most effective knockdown constructs in A549 cells (sh2 and sh3) were evaluated. Treatment of A549 cells with 20****µM cisplatin induced accumulation of p53 and the pro-apoptotic p53 target, Bax, and decreased accumulation of the anti-apoptotic protein Bcl-2, consistent with the DNA damage induced intrinsic apoptotic pathway (Figure 4A). Expression of these apoptotic regulators was unaffected by knockdown of CLPTM1L alone. However, the anti-apoptotic protein Bcl-xL, while unaffected by cisplatin treatment in A549 cells, was decreased when CLPTM1L levels were squelched by shRNA expression. Knockdown of CLPTM1L with shRNAs reduced Bcl-xL protein levels by 81% and 76% in cisplatin treated cells (p<0.003, Two-tailed Student’s T-Test) as measured by three independent Western analyses (Figure 4B). We therefore stably expressed exogenous Bcl-xL in cells with and without knockdown of CLPTM1L to evaluate the role of Bcl-xL in CLPTM1L protection from apoptosis. Re-expression of Bcl-xL was confirmed by Western blot (Figure 4C). While knockdown of CLPTM1L increased apoptosis in empty vector transfected cells by 71%, exogenous Bcl-xL eliminated the CLPTM1L dependent apoptosis (Figure 4D). Resistance to genotoxic stress induced apoptosis closely corresponded to Bcl-xL levels, and Bcl-xL reconstitution completely abrogated sensitization to genotoxic stress induced apoptosis by CLPTM1L.

## Discussion

Survival of cells undergoing DNA damage from genomic instability or genotoxic stress leads to the accumulation of genetic lesions characterizing human tumors. Abrogation of cell-cycle checkpoint and apoptotic safeguards against replication of damaged DNA is a hallmark of cancer. The result of protection against DNA damage induced apoptosis includes both vulnerability to unchecked somatic mutation and decreased sensitivity to radiotherapy and chemotherapy. The previously referenced study by Zienolddinny et al. suggests that CLPTM1L polymorphisms may affect DNA damage accumulation [4]. Based on our observations, it is plausible that protection from apoptosis by CLPTM1L contributes to accumulation of DNA damage, thereby conferring susceptibility to tumorigenesis. An alternative hypothesis is that CLPTM1L plays a role in recognition or repair of DNA damage, affecting the accumulation of such damage. Another is that CLPTM1L directly influences the amount of DNA damage that is incurred by genotoxic agents. Our data (Figure S4) suggests that CLPTM1L expression does not directly affect levels of acute DNA damage incurred by cisplatin or NNK. Moreover, the current study demonstrates that CLPTM1L has an apoptotic role downstream of DNA damage and through regulation of Bcl-xL expression, which is likely to affect accumulation of DNA damage. The observation of differential accumulation of Bcl-xL upon modulation of CLPTM1L expression, along with reconstitution of an apoptosis resistant phenotype with expression of exogenous Bcl-xL, provide evidence that CLPTM1L acts upstream of Bcl-xL to confer resistance to genotoxic stress induced apoptosis. In exogenous Bcl-xL expression experiments, it appears that less Bcl-xL is expressed in vector containing cells than was observed in previous experiments, although decreased accumulation in cells with CLPTM1L knockdown remains evident. Concurrently, apoptotic killing is slightly more robust in vector containing cells observed in previous experiments, but the trend of increased sensitivity upon loss of CLPTM1L and Bcl-xL remains. Importantly, this study shows that the anti-apoptotic function of CLPTM1L is not exclusive to cisplatin sensitivity, but is a general inhibition of the mitochondrial pathway of apoptosis.

Common overexpression of CLPTM1L in tumors supports the notion of an oncogenic or tumor promoting role for CLPTM1L in lung cancers. Protein expression studies performed and annotated by the Human Protein Atlas project [24] confirm our findings. Immunohistochemistry on human normal alveolar cells and lung tumors demonstrated negative expression of CLPTM1L in normal tissues, while expression in 12 lung tumors averaged a score of 1.91 on a scale of 0–3 (Figure S5). A score of 0 is negative, 1 is “weak”, 2 is “moderate” and 3 is “strong”. Six of these patients were diagnosed with squamous cell carcinoma and six were diagnosed with adenocarcinoma. No significant difference in expression between the two pathologies was observed. A total of 66 normal tissues from various organs scored an average of 0.98 (weak). None of the lung tumors tested were negative. Lung tumor cell lines A549 (NSCLC) and SCLC-21H (small cell) showed strong staining and moderate staining, respectively.

Microarray data from tumor and immortalized cell lines demonstrates that mechanisms other than copy number gain result in increased expression in a subset of tumors. In genome wide associations, the 5****p15.33 locus accounts for the greatest contribution to lung cancer risk of three known loci in humans [6]. The 5****p locus is also implicated in cutaneous squamous cell carcinoma and melanoma [25], ovarian cancer [26], testicular germ cell cancer [27] and cervical cancer by copy number gain [8]. In the cervical cancer study, CLPTM1L emerged from the 5****p locus as having expression patterns that correlated with copy number gain. Another recent study of copy number and expression changes in cervical cancer cell lines revealed that with copy number gain across 5****p, CLPTM1L expression was increased approximately 5 fold over normal cervical epithelial cells, while expression of the other genes at 5****p15.33 was not changed [9]. The TERT gene is also within this genetic region. In analysis of SNPs in the 5****p region, we have found that lung cancer associated SNPs are not associated with telomere length (data not shown), in agreement with two other studies [28], [29]. In contrast, a study by Rafnar et al. showed an association (*p* = 0.017 and 0.027, respectively) between 5****p variants (rs401681 and rs2736098) and telomere length, although this effect was only seen when women older than 75 years with homozygous genotypes were included [30]. To our knowledge, no common coding mutations in either CLPTM1L or TERT have been identified. It is plausible that both TERT and CLPTM1L contribute to susceptibility at this locus, having a co-founder effect. A recent study [5] provides multiple lines of evidence that the lung cancer association of rs31489 in the CLPTM1L gene is an independent observation from the association of rs2376100 in the TERT gene. The most recent dense genotyping study of 5****p15.33 found multiple associations at 5p15.33, with the region of association being centered over CLPTM1L [7]. Our previous analysis shows that rs31489 is the variant most strongly associated with familial lung cancer at this locus. The current study indicates that CLPTM1L also plays an important functional role in relation to cancer, and that this gene may to be at least partially responsible for the association of variants in the 5p15.33 region with lung cancer. Continued efforts to further define the genetic variation driving lung cancer susceptibility in this genetic region are an important next step in evaluating the relationship of CLPTM1L and other genes in the region with lung tumor susceptibility.

Common overexpression of CLPTM1L in lung tumors and a functional role in genotoxic stress induced apoptosis identify CLPTM1L as an important factor influencing survival of DNA damaged tumor cells and potentially lung cancer susceptibility. Targeting CLPTM1L as well as Bcl-xL may prove to be useful approaches to chemoprevention and lung cancer therapy. Targeting these anti-apoptotic proteins may also have potential for sensitization of tumors to traditional chemotherapies and radiotherapies.

## Materials and Methods

### RT-Quantitative Real-Time PCR

Patient matched tumor and tumor-adjacent normal RNA samples were obtained from the Tissue Procurement Core at Washington University in St. Louis under protocol approved by the Institutional Review Board at Washington University in St. Louis School of Medicine, Human Research Protection Office. Written consent was obtained from all patients participating in this tissue bank. RNA was isolated from cell lines using Tri-zol reagent and protocols (Invitrogen, Carlsbad, CA). Quantitative real-time PCR (qPCR) was conducted using the method as described previously (Chaparro, Wen et al. 2005). Briefly, one microgram of total RNA per sample was converted to cDNA using the SuperScript First-Strand Synthesis system for RT-PCR (Invitrogen, Carlsbad, CA). Quantitative RT-PCR assay was done using the SYBR Green PCR Master Mix (Applied Biosystems, Foster City, CA). One microliter of cDNA was added to a 25 µL total volume reaction mixture containing water, SYBR Green PCR Master Mix, and primers. Each real-time assay was done in duplicate on a BioRad MyIQ machine. Data were collected and analyzed with Stratagene Mx3000 software. The *β-actin* gene (Actb) was used as an internal control to compute the relative expression level (ΔC_T_) for each sample. Primer set efficiency and linearity was calculated, and normalization was performed in accordance with MIQE guidelines. The fold change of gene expression in tumor tissues as compared to the paired normal tissues was calculated as 2^d^, where d  =  ΔC_T__normal_ – ΔC_T tumor_.

### Microarray Analysis

Expression microarrays were performed using the Illumina HumanWG-6 V3 platform, which contains 48,800 probes corresponding to 20,700 unique genes. RNAs (500 ng) were labeled and hybridized to the BeadChip arrays as specified by the manufacturer (www.illumina.com). Array data were pre-processed with the MBCB algorithm (Ding, LH et al, Nucl. Acids Research, 2008, 36:e58) and differential expression was determined by calculating fold change and T test.

### Cell Culture, Knockdown and Overexpression

A549 cells (ATCC, Manassas, VA), recently authenticated by Genetica Laboratores, Inc. (Cinncinati, OH), were cultured in RPMI-1640 plus 10% FBS (Invitrogen, Carlsbad, CA). Cells were transduced with lentiviral short-hairpin RNA (shRNA) vectors based on the pLKO.1 vector and designed to specifically target human CLPTM1L transcript (Sigma, St. Louis). Empty vector or vector knocking down CLPTM1L transcript were first packaged in 293T cells (Orbigen, San Diego, CA) with helper plasmids and then transduced into A549 cells with 8 µg/ml Polybrene (Sigma, St. Louis, MO). Media was replaced 24 hours after transduction, and cells were split 1∶4 48 hours after transduction. At 72 hours post transduction, cells harboring lentiviral constructs were selected with 1 µg/ml puromycin for 2–4 days, until mock infected cells were dead. Surviving cells were pooled.

pBABEpuro expression vector or pBABEpuro:CLPTM1L, cloned by PCR from pOTB7-CLPTM1L, (Thermo Scientific), into EcoRI/SalI sites of pBABE-puro, was transfected into H1299 cells. Transfected cells were selected with puromycin until mock transfected cells were dead.

pSSFV Bcl-xL expression vector (Addgene plasmid 8749) or empty vector was transfected into cells with and without knockdown of CLPTM1L as described above. Transfected cells were selected with Geneticin (Invitrogen, Carlsbad, CA).

### Viability Assay

Cells stably expressing shRNA vectors as described above were seeded onto 12-well tissue culture dishes at equal densities of approximately 50% in triplicate. After attachment overnight followed by 48 hours of treatment with the indicated concentrations of cisplatin (Sigma, St. Louis, MO) dissolved in media, camptothecin (Sigma, St. Louis. MO) dissolved in DMSO (Sigma, St. Louis, MO), or DMSO solvent alone. DMSO concentrations in culture media were kept consistent and were at or below 0.08%. Cells were assayed for viable cell numbers by trypan blue staining and counted on a Countess automated cell-counter (Invitrogen, Carlsbad, CA). For camptothecin assays, cell viability was measured by Cell Titer 96 Aqueous One Solution Cell Proliferation Assay (MTS) on 24 well tissue culture plates in triplicate. P-values were determined by one-tailed Student’s T-Test.

### Flow Cytometry

The indicated cell lines were treated for 24 hours with the indicated concentrations of cisplatin on 10 cm tissue culture dishes. 10^6^ cells were washed and suspended in PBS and stained with Annexin V-FITC. Cells in staining solution were diluted with 500****µl PBS and analyzed on a Coulter flow cytometer.

### Caspase 3/7 Assay

The indicated cell lines were plated at densities of 5×10^4^ cells per well of a 12 well tissue culture plate and treated as indicated with genotoxic agents after attachment overnight. Caspase 3 and 7 activity was measured using SensoLyte® Homogeneous Rh110 Caspase - 3/7 Assay Kit (Anaspec, Fremont, CA).

### Western Blotting

The indicated cell lines were treated with cisplatin at the indicated concentrations on 6-well plates for 72 hours. Cells were lysed with 100 µl of 1X NP40 lysis buffer containing proteinase inhibitors, sheared 10 times with a 28 gauge needle, spun at 16,000×g for 30 minutes, normalized by protein concentration as determined by the Bradford method, and the supernatant boiled for 10 min. 20 µl of normalized lysate was resolved by SDS-PAGE and immunoblotting analyzed with indicated antibodies. The following antibodies were used: rabbit anti-CLPTM1L (Sigma, St. Louis, MO), rabbit anti-beta tubulin (Sigma, St. Louis, MO), mouse anti-Bcl2 clone 124 (Dako, Carpinteria, CA), rabbit anti-Bax #2774 (Cell Signaling, Boston, MA), mouse anti-p53 (Ab-1) (Oncogene, San Diego, CA), Bcl-xL – rabbit Bcl2L1 (AbCam, Cambridge, MA). Quantitation of Western analyses of three independent cultures was done using Image J software available online from NIH at http://rsbweb.nih.gov. P-values determined by one-tailed student’s T-Test.

### COMET Assay

The gel electrophoresis based method for detection of DNA damage was conducted was modified from Olive, et al. *Nature Protocols* **1**, 23 – 29 (2006). Cells were treated with indicated concentrations of NNK, cisplatin or DMSO solvent 0.1% for 24 hours. Microscope slides were pre-coated with low melting point agarose (Sigma, St. Louis, MO) 1% in dH2O and dried. One mL of low melting point agarose 1% in dH2O kept at 40°C was added to 0.4 mL of a suspension of 2×10^4^ cells/mL in cold media. The agarose/cells mix (1mL/slide) was spread on the pre-coated slides. They were then placed in a lysis solution (2.5****mM****NaCl; 0.1****mM EDTA; 10****mM Tris pH 10, Triton X-100 1%) at 4°C for 2 hours, washed 3×20 min. in electrophoresis solution (300****mM****NaOH; 1****mM EDTA,pH >13), and transferred to an electrophoresis tank in a cold chamber containing electrophoresis solution. A current of 0.45 V/cm was applied for 25 min. The slides were then neutralized with 3×5 min washing steps with TE buffer pH 7.5, and then stained with SYBR Gold (Molecular Probes, Eugene, OR) in TE buffer. At least twenty-five consecutive nucleoids in the centre of each slide were photographed and analyzed for tail DNA content using CASP software. Results are expressed as the Olive Tail Moment.

### Statistical Analysis

Correlation coefficient between percent knockdown and apoptosis was determined using the equation:
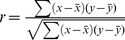
where x and y are the sample means average percent knockdown and average apoptotic index. P-values for correlations were determined using : Soper, D.S. (2012) "p-Value Calculator for Correlation Coefficients (Online Software)", http://www.danielsoper.com/statcalc3

## Supporting Information

Figure S1 A) Scatter plot of fold change in TERT expression in tumors over paired normal tissues on the x-axis, vs. the same for CLPTM1L expression on the y-axis. B) Scatter plot of relative TERT expression in tumor cell lines on the x-axis vs. relative CLPTM1L expression on the y-axis. Relative expression normalized to the average.
(TIFF)

Figure S2 Cell viability as measured by MTS assay and expressed as absorbance at 490 nm of A549 cells with CLPTM1L knockdown after 48 hours with a range of doses of camptothecin.
(TIFF)

Figure S3 (A) Scatter plot of percent knockdown of CLPTM1L in assayed A549 cells versus percent of cells staining positive for Annexin V as measured by flow cytometry. (B) Scatter plot of percent knockdown of CLPTM1L in assayed H838 cells versus Caspase 3/7 activity relative to vector control as measured by colorimetric Caspase 3/7 assay. Trend lines and r^2^ values were added using Microsoft Excel and as describe in the methods section.
(TIFF)

Figure S4 (A) Representative micrographs of COMET assay for DNA damage in cells with CLPTM1L knockdown under 0 or 100 µM NNK. (B) DNA damage defined by the average Olive tail moment under NNK or cisplatin treatment.
(TIFF)

Figure S5 Annotated immunohistochemistry showing CLPTM1L expression in normal and lung tumor tissues and lung tumor cell lines from the Human Protein Atlas. Staining was scored as follows: 0 =  negative, 1 =  weak, 2 =  moderate, 3 =  strong.
(TIFF)

Table S1 Characteristics of the study population used for matched tumor and normal lung tissue expression studies.
(TIFF)
