# Supplementary figures and images for: Functional Characterization of CLPTM1L as a Lung Cancer Risk Candidate Gene in the 5p15.33 Locus
Source: PLoS One. 2012 Jun 4;7(6):e36116. doi: 10.1371/journal.pone.0036116 (PMC3366984; doi:10.1371/journal.pone.0036116)

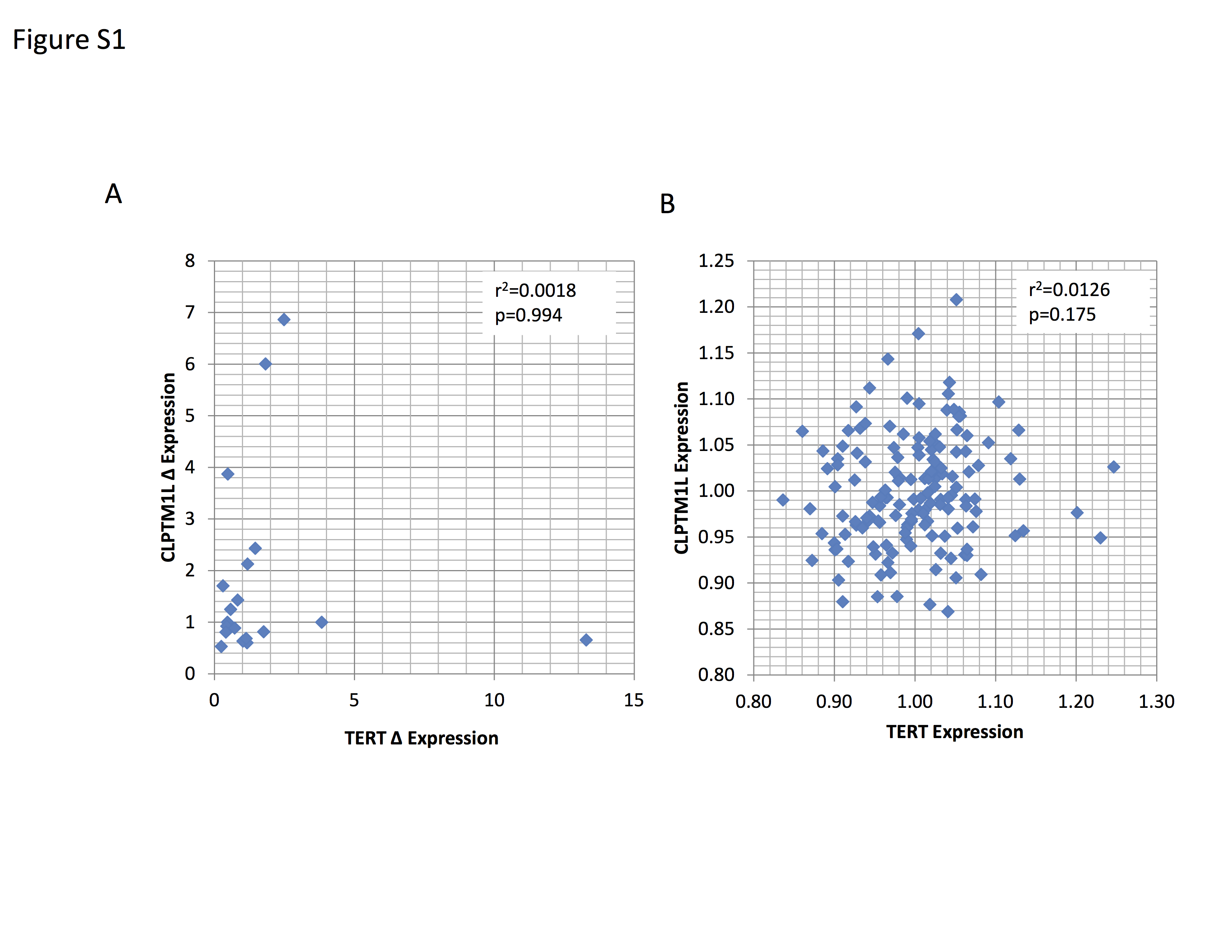

Supplement: Figure S1 — A) Scatter plot of fold change in TERT expression in tumors over paired normal tissues on the x-axis, vs. the same for CLPTM1L expression on the y-axis. B) Scatter plot of relative TERT expression in tumor cell lines on the x-axis vs. relative CLPTM1L expression on the y-axis. Relative expression normalized to the average. (TIFF) [file pone.0036116.s001.tif]

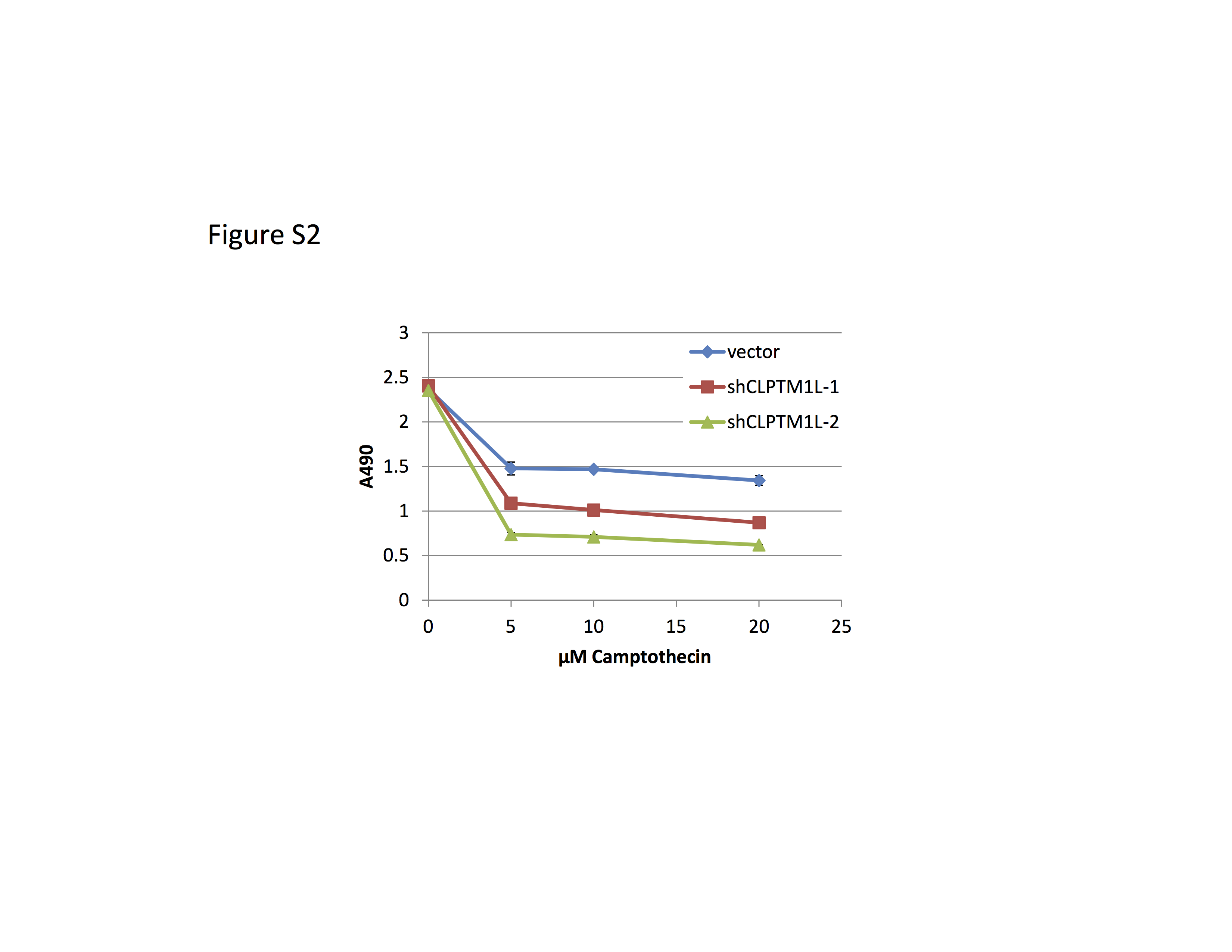

Supplement: Figure S2 — Cell viability as measured by MTS assay and expressed as absorbance at 490 nm of A549 cells with CLPTM1L knockdown after 48 hours with a range of doses of camptothecin. (TIFF) [file pone.0036116.s002.tif]

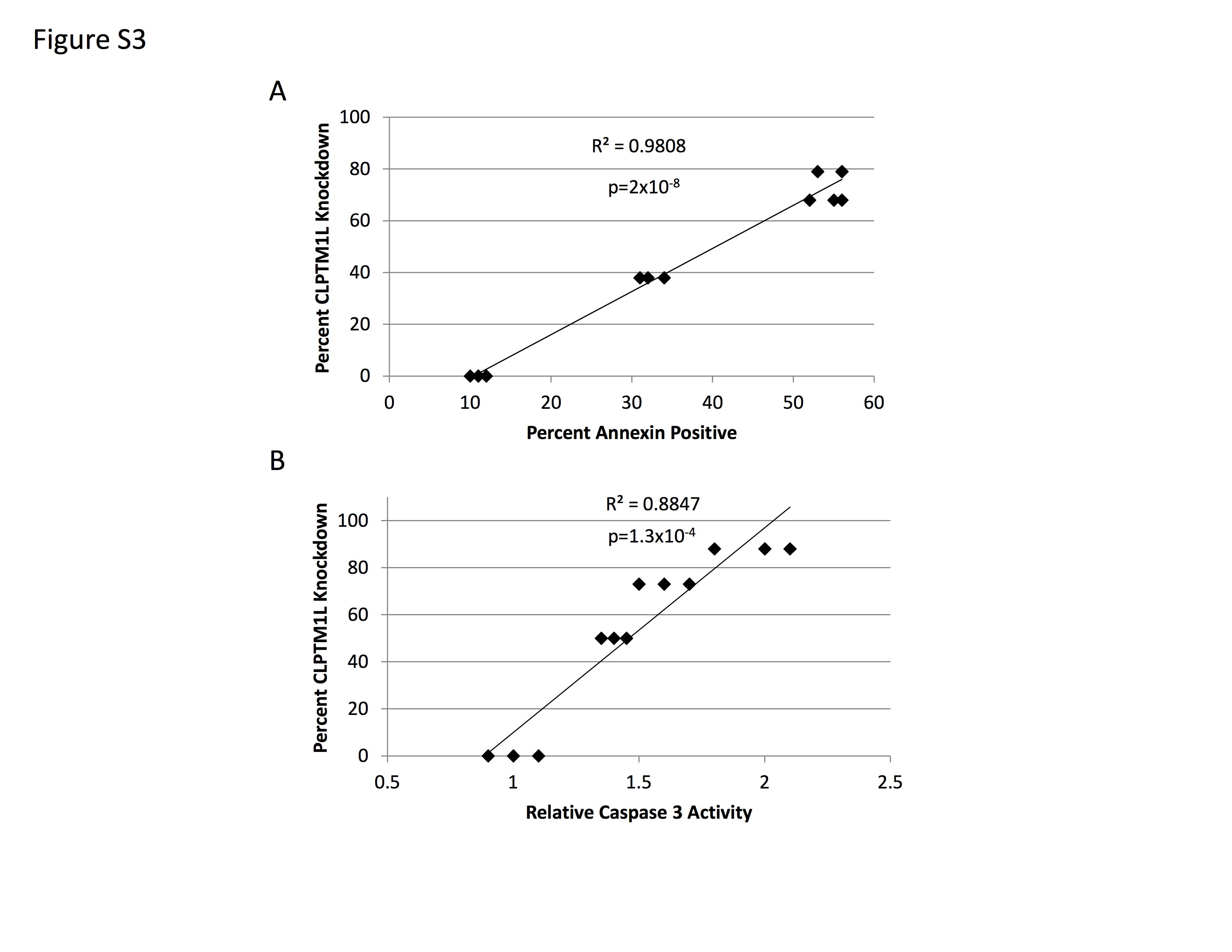

Supplement: Figure S3 — (A) Scatter plot of percent knockdown of CLPTM1L in assayed A549 cells versus percent of cells staining positive for Annexin V as measured by flow cytometry. (B) Scatter plot of percent knockdown of CLPTM1L in assayed H838 cells versus Caspase 3/7 activity relative to vector control as measured by colorimetric Caspase 3/7 assay. Trend lines and r2 values were added using Microsoft Excel and as describe in the methods section. (TIFF) [file pone.0036116.s003.tif]

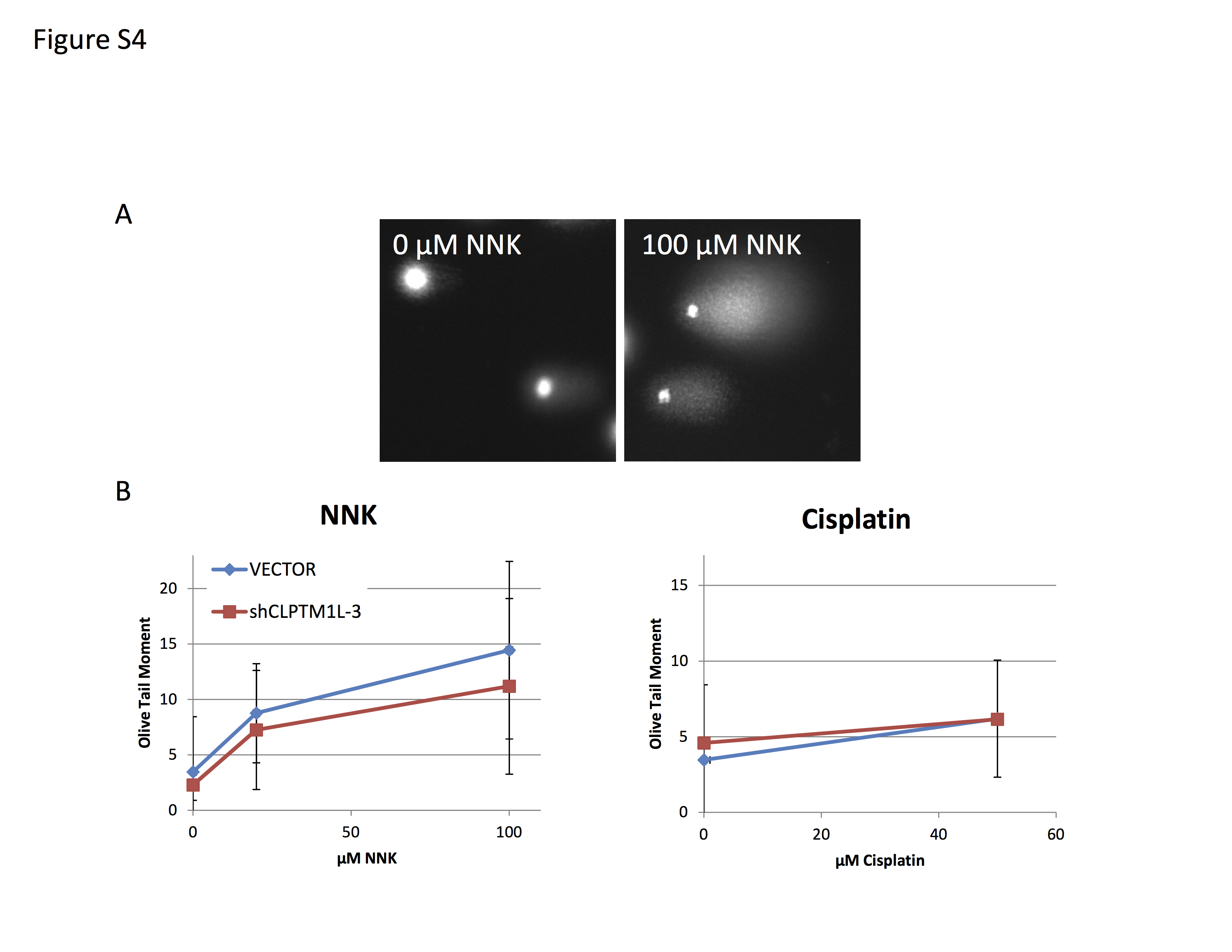

Supplement: Figure S4 — (A) Representative micrographs of COMET assay for DNA damage in cells with CLPTM1L knockdown under 0 or 100 µM NNK. (B) DNA damage defined by the average Olive tail moment under NNK or cisplatin treatment. (TIFF) [file pone.0036116.s004.tif]

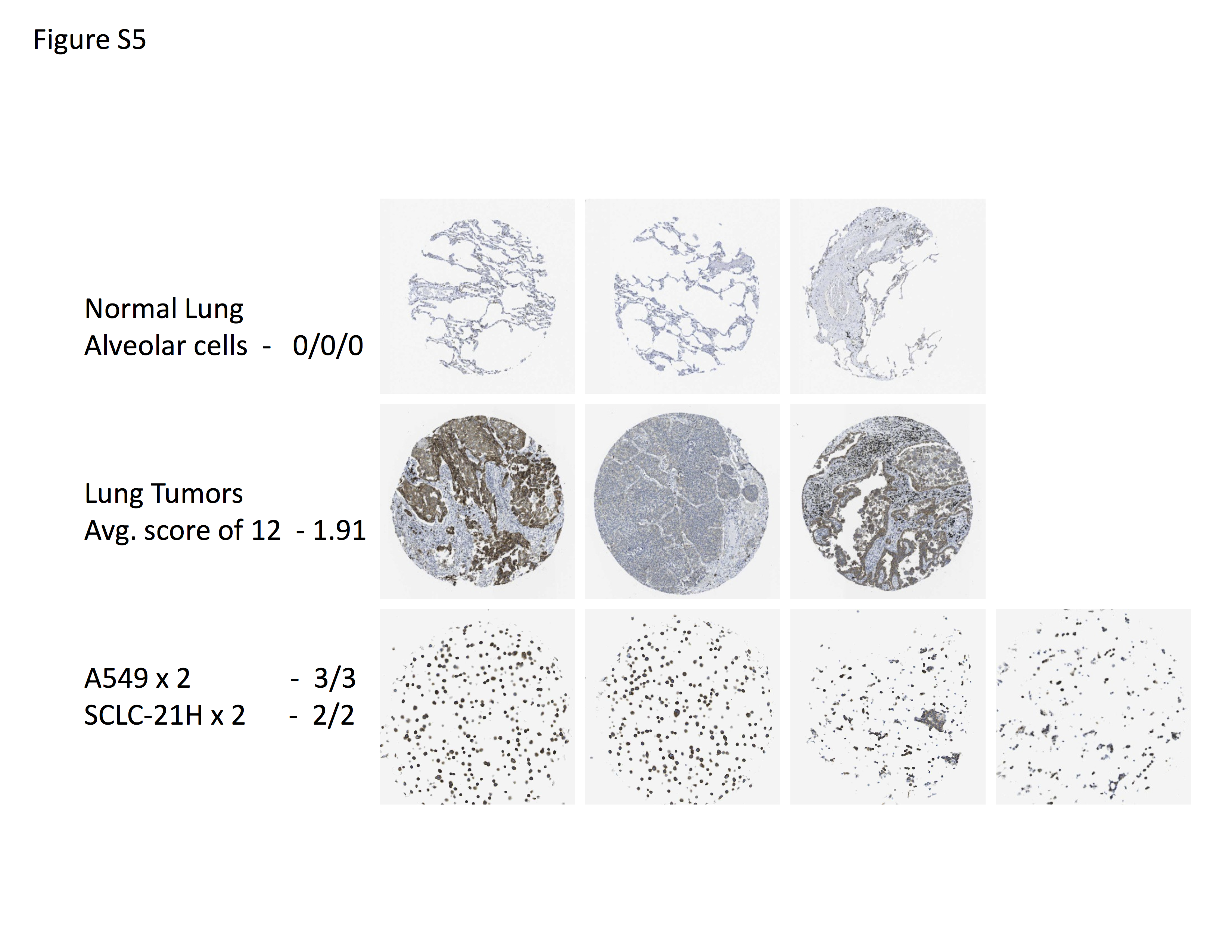

Supplement: Figure S5 — Annotated immunohistochemistry showing CLPTM1L expression in normal and lung tumor tissues and lung tumor cell lines from the Human Protein Atlas. Staining was scored as follows: 0 = negative, 1 = weak, 2 = moderate, 3 = strong. (TIFF) [file pone.0036116.s005.tif]

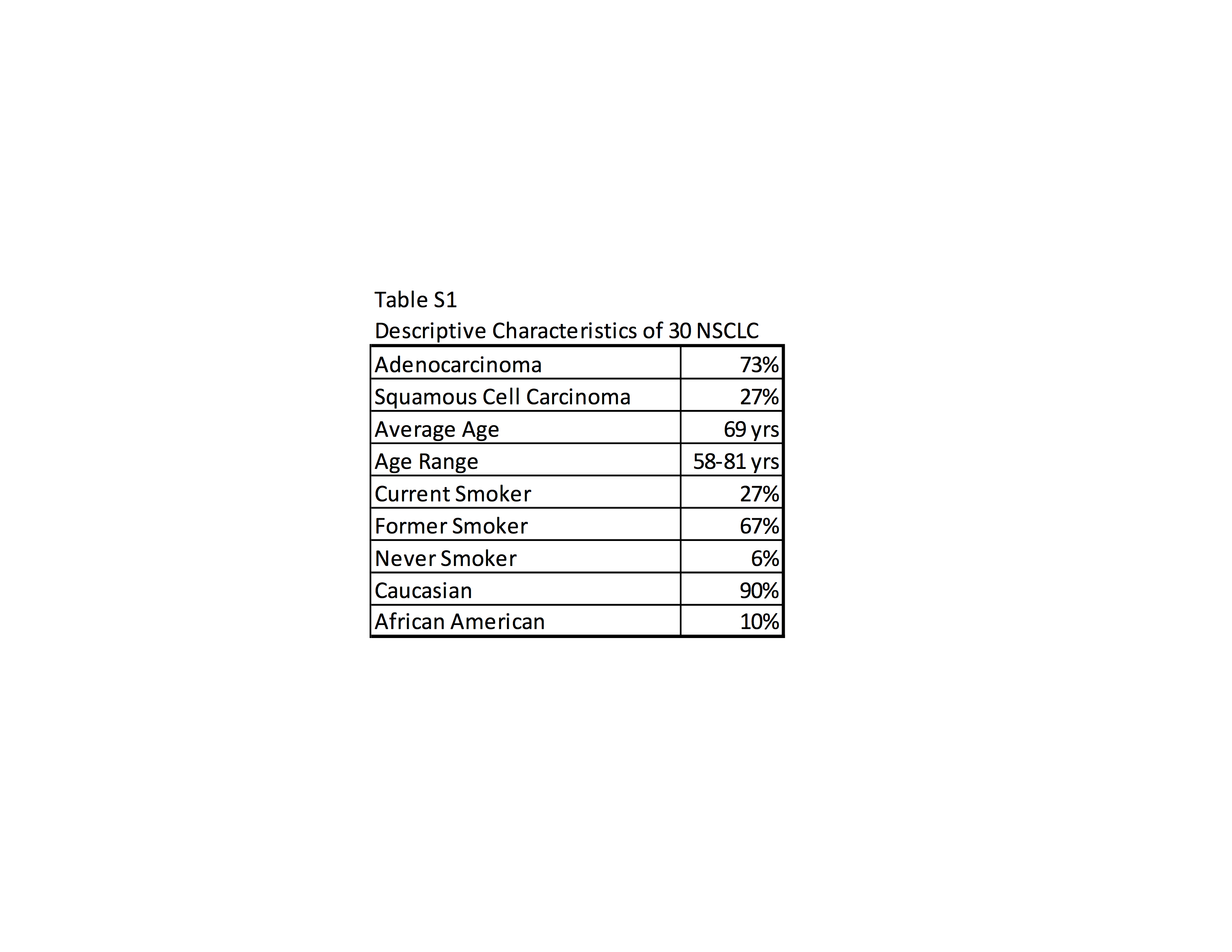

Supplement: Table S1 — Characteristics of the study population used for matched tumor and normal lung tissue expression studies. (TIFF) [file pone.0036116.s006.tif]
